# Influence of mobile phone calls on the compliance of the recommended four antenatal care visits in Kisii County, Kenya: a cluster randomized control trial

**DOI:** 10.4314/ahs.v24i4.23

**Published:** 2024-12

**Authors:** Zillah M Malachi, Lucy W Kivuti-Bitok, Anna K Karani, Joyce J Cheptum

**Affiliations:** 1 Department of Nursing, School of Health Sciences, Kisii University, Kenya; 2 Department of Nursing, College of Health Sciences, University of Nairobi, Kenya; 3 Kenyatta National Hospital; 4 Aga Khan University, Nairobi

**Keywords:** Influence of mobile phone calls, four antenatal care visits, Kisii County, Kenya

## Abstract

**Background:**

Antenatal care attendance is still low in sub-Saharan countries. While mobile phones have shown to improve outcomes in maternal health services, there are few published studies on the use of mobile phone calls in antenatal care.

**Objective:**

To determine the influence of mobile phone calls on pregnant women's completion of the recommended 4 ANC visits in Kisii County, Kenya.

**Methodology:**

16 sub county health facilities (clusters) were randomly assigned to either intervention or routine care. A total of 160 pregnant women were recruited in their first antenatal care visit and followed up until delivery. The intervention involved calling mothers through their mobile phones to give health education on antenatal care every month until delivery. The primary outcome measure was the completion of the 4 recommended antenatal care (ANC) visits while secondary outcome measures were; women receiving iron and folate supplements, and completion of all recommended laboratory tests.

**Results:**

50% of the women in the intervention group and 35% in the control group completed the four recommended ANC visits. The intervention was not a significant predictor of women receiving iron and folate supplements, RR, 1.07 (0.93 – 1.25), p – value = 0.412. However, the intervention was associated with a 46% increase in women completing all required antenatal care laboratory investigations.

**Conclusion:**

The use of mobile phone calls in antenatal health education show promise in improving antenatal care attendance among pregnant women.

## Introduction

The number of antenatal care visits is a good indicator of the utilization of these services and every visit counts^1^. The World Health Organization (WHO) now recommends eight contact visits with a health care provider to minimize morbidity and mortality associated with pregnancy^2^. However, research shows that antenatal care attendance is still low in developing countries especially the sub Sahara Africa where about 53% of the mothers complete the previously recommended four Antenatal Care (ANC) visits^3^. Most of the developing countries including Kenya are yet to adopt the new WHO recommendation of eight ANC visits.

Pregnant mothers face several challenges during the antenatal period ranging from lack of knowledge of antenatal care services offered and accessibility of these services^4^,^5^. There are assumptions that women who attend antenatal care clinics receive health education on pregnancy issues, but studies have shown that these women may not receive adequate health information^6^,^7^. There is evidence that health education improves the utilization of antenatal care services^8^,^9^.

In Kenya, the Kenya Demographic Health Survey of 2014 indicated that approximately 50% of pregnant women attend antenatal care clinics^10^. The report further stated that despite this attendance, only 58% of these mothers completed four ANC visits.

The utilization of mobile phones in communication is increasingly becoming popular and may be used to facilitate health education and hence improve health service utilization^11^. By the end of 2025, it is estimated that more than half of the world population will be living within the reach of a 5G network^12^. Many studies have explored the use of short message services using mobile phones in the provision of health education and also reminders to take medication or to attend antenatal care clinics. In Kenya, mobile phones have been used to provide health information through one- or two-way short message services. However, few experimental studies have explored the influence of phone calls in antenatal care health education. The present study sought to examine the influence of antenatal health education through mobile phone calls on women's compliance with the four recommended antenatal care visits in Kisii County, Kenya. We hypothesized that the intervention group would comply with the recommended four antenatal care visits than the control group.

## Methods

### Design

This study was a cluster randomized control trial with sub county health care facilities being the unit of randomization.

### Setting

Kisii County consists of nine sub-counties, eight of which are rural and one is an urban area.

### Inclusion and exclusion criteria

The study targeted pregnant women attending ANC clinics in rural health centers. Anecdotal data shows that utilization of maternal health services is low in rural areas. Sixteen rural sub county health care facilities which registered approximately 20 new pregnant women per month were included in the study. The health care facilities were mapped using the global position system to determine the distance between them. The facilities had to be at least 5 km apart to be eligible to be included in the study. This was to prevent contamination arising from the pregnant women interacting with one another or crossing over to the clinics for antenatal care services.

We enrolled women who were either in their first or second trimester, attending antenatal care clinic for the first time during the current pregnancy. Pregnant women without mobile phones, temporary residents and those with cormodities were excluded.

### Sample size

Based on available resources, a total of 160 pregnant women participated in the study at a ratio of 1:1 with 80 participants in each of the study arms. The sample size was deemed sufficient to detect a 25% increase in the proportion of pregnant women completing at least four ANC visits from 30% according to the unpublished local Health Information System reports for the year 2019, Ministry of Health, Kisii County, Kenya to 55%. We assumed a 95% confidence level and a power of 80%. An intraccluster correlation coefficient of 0.02 as per a Cochrane review on antenatal care coverage^14^ was also considered. We calculated the sample size for binary outcomes as proposed by^15^.

### Outcome measure

The primary outcome of interest was at least 4 ANC visits completed while the secondary outcome measures were; number of women receiving prophylaxis for anaemia (iron and folate supplements) and completion of all the recommended laboratory tests during pregnancy.

### Intervention

The intervention involved calling the women through the mobile phones every month to give antenatal health education until they delivered. The message content passed to the participant was as per the Kenya Ministry of Health antenatal care guideline^16^. These included; Mother's birth plan, education on danger signs and emergency preparedness, the importance of preventive services, nutrition, self-care, exercise, and rest. The duration of the call was 20 minutes on average per participant.

### Routine care

This entailed women receiving usual antenatal care at the health centers every month as per the Ministry of Health, Kenya guidelines.

### Randomization and allocation

We performed simple random sampling using the lottery method to randomly assign the participating rural public health facilities to either routine care (n = 8) or mobile phone call health education intervention (n=8). Due to the nature of the intervention, blinding/masking was not done for participants and research assistants.

### Data collection procedures

Eligible pregnant women were enrolled between February 2020 and May 2020. All women who were enrolled received routine antenatal care in the participating health centers. The women in the intervention group received mobile phone calls on antenatal health education every month in addition to routine care. In each participating sub county health centre, one nurse attending to the pregnant women in the ANC clinic was recruited as a research assistant. These nurses in the intervention arm sites received training on mobile health education intervention. A questionnaire was used to collect baseline data on sociodemographic characteristics. Another questionnaire was administered at the end of the study to determine the uptake of antenatal care services. The data was cleaned, coded, and entered into an excel sheet.

We visited the health centers every week to ensure the quality of the data collection process and implementation of the intervention. The research assistants were facilitated with airtime to enable them make calls to the pregnant women. All the women participating in the study were contacted at term to remind them to call the nurses immediately after delivery so that the post-intervention interview could be conducted.

### Data analysis

All collected data were transferred to R, version 4.2.0. (2022) from excel for analysis. Analysis of the data was done at the individual level. We used the chi-square test of association to determine the homogeneity of baseline data in the two groups. Univariate and multivariate logistic regression were used to determine the odds of mothers completing at least four antenatal care visits in both groups while the Wilcoxon sum rank test was used to determine the association between the intervention and the number of ANC visits. We computed the risk ratios of both the primary and secondary outcome variables to determine the effect of the intervention. The level of significance was set at p < 0.05 for these analyses.

### Ethical considerations

Ethical approval to conduct this study was given by the Institutional Review Board (IRB) of the Kenyatta National Hospital/University of Nairobi, Ethics and Research Committee, Ref. No. KNH – ERC/A/335 and National Commission for Science Technology and Innovation (NACOSTI), Ref. No. 183437. Approval from the participating health centers was sought by writing formally to the heads of the health facilities. The study objectives, risks and benefits of the study were explained to all eligible women. The process of data collection was explained to the participants and they were assured of privacy and confidentiality and informed that participation was voluntary. In addition, they were informed that they could withdraw from the study at any stage during the study. A verbal and written consent were obtained from the participants.

## Results

**Figure 1:**
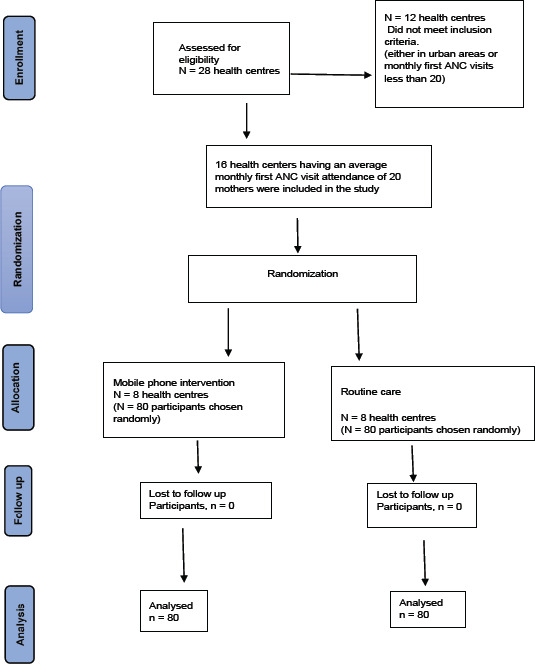
CONSORT flow diagram for the study

The total number of eligible participants recruited into the study was 160 i.e., 80 participants in each arm as shown in Figure 1. There were no losses to follow up.

### Sociodemographic characteristics of the study participants

Most of these women were aged between 20 and 30 years, 52(65%) in the control group and 54(67.5%) in the intervention group. The majority of women in both groups were married, had between one and two children, and had at least a secondary school education. Most of the women in the two groups made their first visit in the second trimester. There were no significant differences in the sociodemographic characteristics between the two groups as shown in table 1.

**Table 1:** Comparison of the sociodemographic characteristics between the intervention and control group

Variable	Control group N = 80 N(%)	Experimental N = 80 N(%)	OR (95% CI)	p-value
**Age**				0.51
**< 20 years**	17(21.2)	15(18.8)	Ref	
**20 – 30 years**	52(65.0)	54(67.5)	1.17(0.53 – 2.63)	
**30 – 40 years**	7(8.8)	10(12.5)	1.60(0.48 – 5.53)	
**>40 years**	4(5.0)	1(1.3)	0.32(0.01 – 2.62)	
**Level of education**				0.49
**Primary**	20(25)	22(27.5)	Ref	
**Secondary**	46(57.5)	39(48.8)	0.77(0.36 – 1.63)	
**Tertiary**	14(17.5)	19(23.8)	1.23(0.49 – 3.14)	
**Previous abortions**				0.21
**No**	68(85.0)	74(92.5)	Ref	
**Yes**	12(15.0)	6(7.5)	0.47(0.15 – 1.29)	
**Gestational age at 1^st^ visit**				0.99
**>16 weeks**	53(66.2)	54(67.5)	Ref	
**<16 weeks**	27(33.8)	26(32.5)	0.95(0.49 – 1.84)	
**No. of children**				0.93
**None**	32(40)	29(36.2)		
**1 – 2**	36(45)	38(47.5)		
**3 – 5**	12(15)	12(15)		
**>5**	0	1(1.3)		
**Marital status**				0.83
**Married**	66(82.5)	68(85)		
**Single**	14(17.5)	12(15)		

### Association of the intervention with the primary and secondary outcome variables

Comparing the groups, most women in the intervention group completed at least four ANC visits, 40(50%) as compared to the control group 28(35%). This difference was not statistically significant with a chi-square test, χ2 = 3.0946, df = 1, p = 0.079. The average number of ANC visits attended in both the intervention and control groups was 3, with a median of 3.0 and an interquartile range (IQR) of 3 to 4. Further evaluation of the effect of the intervention on the number of visits completed using a Wilcoxon rank-sum test showed a significant result of; (W = 3775, p = 0.039).

While the intervention was not a significant predictor of women receiving iron and folate supplementation, it was associated with a 46% increase in women completing all required antenatal care laboratory investigations (p = 0.001) as shown in table 2.

**Table 2:** Influence of the intervention on the primary and secondary outcome variables

Outcome variable	Control n (%)	Intervention n (%)	Risk Ratio (95% CI)	P – value
**Completion of at least 4 antenatal care visits** **Yes** **No**	28 (35) 52 (65)	40 (50) 40 (50)	1.42 (0.98 – 2.07)	0.079
**Received iron and folate supplements** **Yes** **No**	63 (78.7) 17 (21.3)	68 (85) 12 (15)	1.07 (0.93 – 1.25)	0.412
**All ANC investigations done** **Yes** **No**	45 (56.3) 35 (43.7)	66 (82.5) 14 (17.5)	1.46 (1.17 – 1.82)	0.001

### Association of the primary outcome variable and other factors

The univariate and multivariate logistic regression analysis presented in table 3, shows that education level, p = 0.021, history of previous abortions, p <0.001, and gestational age at the first visit, p<0.001 were significant predictors of mothers completing at least 4 ANC visits.

**Table 3:** Univariable and multivariable logistic regression analysis showing the relationship of the independent variables with completion of at least 4 ANC visits (n = 160, intervention = 80, control = 80)

Variable	<4 ANC visits N =92	>4 ANC visits N = 68	Crude OR (95% CI)	P-value	Adjusted OR (95% CI)	p-value
**Group**				0.056		0.079
**Control**	52(56.5)	28(41.2)	Ref		Ref	
**Experimental**	40(43.5)	40(58.8)	1.86(0.99 – 3.53)		1.85(0.98– 3.52)	
**Level of**				0.007		0.021
**education**	30(32.6)	12(17.7)	Ref		Ref	
**Primary**	49(53.3)	36(52.9)	1.97 (0.88-4.40)		1.82(0.83– 4.17)	
**Secondary**	13(14.1)	20(29.4)	6.14 (2.08-18.11)		3.75(1.44– 10.30)	
**Tertiary**						
**Previous**				0.001		<0.001
**abortions**	90(97.8)	52(76.5)	Ref		Ref	
**None** **Yes**	2(2.2)	16(23.5)	13.85(3.74– 89.80)		12.8(3.43– 90.70)	
**Gestational age**				<0.001		<0.001
**at 1^st^ ANC visit**	86(93.5)	21(30.9)	Ref		Ref	
**>16 weeks** **<16 weeks**	6(6.5)	47(69.1)	32.08(12.93-93.07)		30.5(12.2– 89.70)	

## Discussion

This study assessed the influence of mobile phone calls on pregnant women's completion of the recommended 4 ANC visits. The findings from this study indicate that the intervention increased the proportion of pregnant women completing at least four antenatal care visits by 20%, while the proportion of women receiving routine care increased by 5%. However, this was marginally statistically significant and could have been attributed to the small sample size that was used. Overall, the intervention had a positive association with the total number of ANC visits completed. This might be explained by the differences in gestational age at the time of recruitment because women are required to come back to the clinic every four weeks for antenatal care unless they have complications which require frequent checkups.

A comparable study using similar methodology as the current study conducted in Nigeria assessing the effect of mHealth voice messaging on ANC use in primary health care facilities reported an increase in ANC attendance^15^. In the same vein, a quasi-experimental study carried out in a health centre and dispensary in Dodoma, Tanzania, reported an increase in the utilization of antenatal care services among pregnant women who received mobile phone health education messages and reminders through short message service and phone calls^16^. A systematic review and meta-analysis of mHealth interventions in low resource settings has also shown that mobile health interventions increase the uptake of antenatal care services^17^. However, it is worth noting that there is a slight difference between the current study and the cited studies in terms of study design and mobile phone intervention evaluated. This could probably explain the difference in findings. Nonetheless, the use of mobile phones in antenatal care shows significant improvement in the uptake of services.

Findings from this study indicate that health education through mobile phone calls did not influence the uptake of iron and folate supplementation. This could be explained by the fact that these prophylactic drugs are readily available in all public health facilities in Kenya and can be accessed by pregnant women as long as they attend ANC clinic. Contrary findings were reported in Northern Ethiopia where health education and the number of ANC visits were associated with adherence to iron and folate supplementation^20^. Demographic health surveys conducted in Asia, Africa, Latin America and Caribbean regions show that more ANC visits are associated with increased uptake of iron and folate among pregnant women^21^,^22^. According to these studies, compliance to iron and folate supplementation is low and is associated with low uptake of antenatal care services. However, there is need for more research with regard to the uptake of iron and folate supplementation among pregnant women since there is inconclusive evidence on the enablers and barriers to compliance.

Pregnant women in the current study who attended more ANC visits were more likely to undergo all the tests that are required during pregnancy. Attending more ANC visits ensured that the women received adequate ANC services including laboratory services. Completing four or more ANC visits has been reported as influencing adequate and quality antenatal care including such services as laboratory investigations^23^.

In the current study, other factors contributed to the ANC attendance as well. For example, this study reveals that women who started ANC clinic attendance during the first trimester were more likely to complete the four recommended visits as compared to those who started attending clinic in the second trimester. This is due to the fact that women who attend ANC clinic early during pregnancy have more time for revisits before they give birth as compared to those who initiate clinic attendance late in pregnancy. This result can be corroborated by a review of studies involving 74 studies conducted to determine antenatal care utilization in the Sub Sahara Africa which indicated that early initiation of antenatal clinic attendance influences the total number of ANC visits completed^24^.

It was also evident that women with tertiary-level education were more likely to complete the recommended number of ANC visits. A study conducted in Ethiopia assessing the utilization of antenatal care services in the rural regions also reports similar findings^25^. Probably this could be due to the fact that education equips one with knowledge on various aspects of health and also builds confidence.

The current study demonstrates that history of previous abortions influenced women's antenatal clinic attendance. This probably, could be due to the motivation to prevent future obstetric complications. Similarly, a cross sectional study carried out in rural areas of South Western Ethiopia indicates that women who had an abortion made more ANC visits as compared to those who did not have^25^. The same study also reported that knowledge of danger signs was a significant predictor of ANC utilization. Probably women with a history of abortion are aware of danger signs in pregnancy from experience and this could have contributed to the more ANC visits they made in the current study.

## Limitations

It is worth noting that these results can only be generalized to the community where this study was conducted. In addition, the small sample size may have been inadequate and this could have affected the results.

## Conclusion and recommendations

Based on the findings from the current study and other similar studies, it is evident that mobile phone calls may have a positive influence on antenatal care attendance. Health education provided through mobile phone calls during pregnancy equips the mothers with knowledge of antenatal care on a one-on-one basis, especially on danger signs and pregnancy complications. This enables them to attend ANC clinics to receive services geared towards preventing these complications.

However, there is a need for large sample size studies to evaluate the impact of the intervention on antenatal care service utilization.
